# Clinical and functional outcomes in pediatric patients with Rett syndrome: a 15-year retrospective study

**DOI:** 10.1007/s00431-025-06291-6

**Published:** 2025-07-03

**Authors:** Mariana Cortez Ferreira, Joana De Beir, Maria Inês Barreto, Joana Afonso Ribeiro, Cristina Pereira, Guiomar Oliveira

**Affiliations:** 1 Child Developmental Centre, Hospital Pediátrico de Coimbra, Unidade Local de Saúde de Coimbra, Coimbra, Portugal; 2 Clinical Academic Center of Coimbra, Hospital Pediátrico de Coimbra, Unidade Local de Saúde de Coimbra, Coimbra, Portugal; 3 Faculty of Medicine, University Clinic of Pediatrics, University of Coimbra, Coimbra, Portugal

**Keywords:** Rett syndrome, MECP2, Diagnosis, Follow-up, Children

## Abstract

Rett syndrome (RTT) is a neurological disorder caused by mutations in the *MECP2* gene. It is characterized by apparently normal early neurodevelopment followed by psychomotor regression between 6 and 18 months. There is no cure, and treatment is multidisciplinary, focusing on improving quality of life and functionality. This study aims to characterize RTT patients and evaluate clinical patterns, interventions, and outcomes. A retrospective cohort study was conducted of children diagnosed with RTT and a pathogenic *MECP2* variant, followed at a Portuguese tertiary pediatric hospital from 2010 to 2024. Electronic medical records were reviewed, and epidemiological and clinical data were collected. Twelve RTT patients were followed (91.7% female). The median age at symptom onset was 15 months, and at diagnosis, 35 months. Seven patients (58.3%) had classic RTT. Molecular analysis identified 10 different *MECP2* variants, all de novo mutations. Epilepsy and constipation were the most common comorbidities. Five patients (41.7%) were wheelchair-dependent, and two required non-invasive respiratory support. Almost all patients received physical, occupational, and speech therapies. The median number of chronic medications per patient was two, primarily anticonvulsants. The median number of medical specialties per patient was two, with neuropediatrics and neurodevelopmental pediatrics being the most frequent.

*Conclusions*: RTT prevalence remained stable over 20 years, with a decreased median age at diagnosis, likely due to increased awareness and genetic testing. No clear genotype–phenotype correlation was found. RTT diagnosis remains based on clinical features, particularly neurodevelopmental regression. A multidisciplinary approach was critical, with patients receiving various therapies and support. This study provides valuable insights into RTT in Portugal, contributing to global knowledge.

**Table:** 

**What is known:** • *Rett syndrome is a rare neurodevelopmental disorder primarily affecting females, characterized by regression of motor and communication skills*. • *Limited long-term data exist on clinical progression and functional outcomes in pediatric Rett syndrome*.	
**What is new:** • *A decreasing age at diagnosis was observed, likely reflecting increased awareness and access to genetic testing*. • *In an era of promising clinical trials for potential curative treatments, an individualized, multidisciplinary approach remains the standard of care in most countries, including Portugal*.

**Supplementary Information:**

The online version contains supplementary material available at 10.1007/s00431-025-06291-6.

## Introduction

Rett syndrome (RTT; OMIM entry #312,750) is a progressive, non-degenerative, neurological disorder. First described in 1966 by Austrian pediatric neurologist Dr. Andreas Rett, the causal association between this rare condition and mutations in *methyl-CpG-binding protein 2 (MECP2*) gene was only identified in 1999 [1, 2]. Located on the long arm of the X chromosome (Xq28), it encodes a protein of the same name, which is responsible for the activation and inactivation of other genes, playing a crucial role in the normal maturation and functioning of neuronal cells. Over 250 different mutations in this gene have been described, 99% of which are de novo. The type of mutation and the lyonization of the X chromosome contribute to the variability in the phenotype and severity of this disorder [3–5].

Its classic phenotype is characterized by an initial period of apparently normal growth (except for postnatal deceleration of head growth) and neurodevelopment, followed by a psychomotor development delay or regression between 6 and 18 months of age, with the emergence of the four main clinical criteria of the syndrome: loss of manual skills and language, gait abnormalities, and appearance of stereotypical behaviours [6, 7]. Symptoms progress, and the eventual deterioration will result in individuals being unable to walk, speak, or use their hands functionally [8].

Females are the most frequently affected, with an estimated prevalence of 7.1 per 100,000 females and a cumulative risk of 6.51 per 100,000 person years [1, 9]. The prevalence in males is lower, and they may have a more severe phenotype, with severe postnatal encephalopathy and early childhood death [7, 10]. Males may also have classic RTT due to somatic mosaicism [11, 12].

Despite advances in the diagnosis and treatment of this disorder, the natural history remains complex and with significant interindividual variability [3, 8, 10]. Even though trofinetide (Daybue®) was approved by the Food and Drug Administration (FDA) in 2023 for the treatment of RTT and two gene therapy trials are currently in pivotal trials, no disease modifying treatment currently exists in Europe, particularly in Portugal. Therefore, treatment plans in our country remain multidisciplinary and supportive and are individualized according to the stage of disease evolution and the symptoms present. The primary goals are to improve the quality of life of the patient and their family [3, 8, 10]. Notable therapies include speech, occupational, and physiotherapy, and psychological health support for caregivers and other family members plays an important role in these cases.

In the last 10 years, no studies have been published regarding RTT in Portugal. The main objective of our study is to characterize the population of patients with RTT followed at our university pediatric by examining clinical patterns, the impact of interventions, and functional outcomes. In this way, we aim to contribute to the optimization of provided care, as well as to raise awareness and expand knowledge about this severe neurodevelopmental disorder among pediatricians and other healthcare professionals involved in patient care.

## Materials and methods

### Study design and patient selection

A retrospective cohort study was conducted of children diagnosed with RTT and followed in a reference child development center of a Portuguese University Tertiary Children’s Hospital within the National Health Service from 2010 to 2024. All patients with RTT and a pathogenic *MECP2* gene variant were included. Patients with RTT-like, uncertain genetic diagnosis or with an undefined *MECP2* variant without clinical manifestations were excluded. Clinical data were obtained through electronic medical records review.

### Data collection

The RTT phenotype classification was based on the revised diagnostic criteria of the Rett Search Consortium (Table 1) [7].

**Table 1:** Revised diagnostic criteria for RTT of the Rett Search Consortium [7]

Typical or classic RTT
1. A period of regression followed by recovery or stabilization
2. All main criteria and all exclusion criteria
3. Supportive criteria are not required
Atypical RTT
1. A period of regression followed by recovery or stabilization
2. At least two out of the four main criteria
3. At least five out of the 11 supportive criteria
Exclusion criteria for typical or classic RTT
1. Brain injury secondary to trauma, neurometabolic disease, or severe infection that causes neurologic problems
2. Grossly abnormal psychomotor development in the first 6 months of life
Main criteria for typical or classic RTT
1. Gait abnormalities
2. Stereotypic hand movements
3. Loss of acquired spoken language
4. Loss of acquired purposeful hand skills
Supportive criteria for atypical RTT
1. Abnormal muscle tone
2. Impaired sleep pattern
3. Scoliosis/kyphosis
4. Breathing disturbances when awake
5. Growth retardation
6. Bruxism when awake
7. Peripheral vasomotor disturbances
8. Small cold hands and feet
9. Inappropriate laughing/screaming spells
10. Diminished response to pain
11. Intense eye communication

*RTT* Rett syndrome

Demographics (sex, year of birth), prenatal and perinatal factors (fetal anomalies, type of delivery), neonatal factors (gestational age at birth, birth weight, length and head circumference, Apgar score), psychomotor development milestones (sitting without support, first words, first steps without support), *MECP2* variant, and age at symptom onset, suspected clinical diagnosis, and genetic diagnosis were included in the analysis. Mobility (disability or absence of ability), hand function (loss of hand skills or stereotypic hand movements), communication skills (loss of acquired spoken language, intense eye communication, inappropriate laughing or screaming spells), and the presence of breathing irregularities (breath holding, hyperventilation or forced expulsion of air), bruxism, sleep disturbances, abnormal muscle tone, diminished response to pain, and peripheral vasomotor disturbances or small cold hands and feet were retrieved. Growth retardation was defined as a downward deflection of the growth velocity with the resultant growth curve crossing percentiles [13]. Microcephaly was considered when head circumference was more than two standard deviations below the mean for age and sex [14]. Medical comorbidities (epilepsy, constipation, need for non-invasive respiratory support, scoliosis/kyphosis with or without spinal surgery, gastrostomy placement, strabismus) were recorded even if they resolved over time. Children were considered to have epilepsy if they had unprovoked seizures and an electroencephalogram showing paroxysmal activity or compatible with an epilepsy syndrome [15]. Autism spectrum disorder was considered if the diagnostic tools—Autism Diagnostic Interview-Revised (ADI-R) and Autism Diagnostic Observation Schedule (ADOS)—met the clinical criteria for the diagnosis according to the American Psychiatric Association’s Diagnostic and Statistical Manual, Fifth Edition (DSM-5) [16–18]. Therapies and support measures (physiotherapy, occupational therapy, speech therapy, hydrotherapy, *Snoezelen* therapy, music therapy, hippotherapy, canine-assisted therapy, learning and inclusion measures, National Early Intervention System in Childhood support, and forms of alternative medicine), chronic medication, and specialties involved in follow-up were retrieved. Date and circumstances of death were also included.

### Data analysis

Statistical analysis was performed using the IBM®SPSS® Statistics version 27. Categorical variables are presented as frequencies and percentages. Continuous variables are presented as means and standard deviations (SD) if normally distributed, while non-normally distributed data are presented as medians and interquartile ranges (IQR). Normal distribution was verified through the Kolmogorov–Smirnov test or skewness and kurtosis (maximum tolerated interval of − 1 to 1).

### Ethical approval

Approval was obtained from the Hospital local Ethics Committee (process number PI 2024-ESI.SF-267). All the methods included in this study are in accordance with the declaration of Helsinki.

## Results

Twelve patients with RTT were followed between 2010 and 2024, of which 11 (91.7%) were female. Diagnostic rate was stable during the studied period (Table 1). Baseline characteristics can be found in and detailed individual patient description in Appendix 1.

Only one patient had a prenatal finding of an echographic abnormality (8.3%)—an isolated finding of a single umbilical artery—and one patient was born prematurely (8.3%). All patients had a good adaptation to extrauterine life without the need for life-saving measures. Further description of all baseline and prenatal characteristics, as well as psychomotor development milestones, can be assessed in Table 2.

**Table 2:** Characteristics of the overall sample

	All infants (*n* = 12)
Prenatal and perinatal characteristics	
Full pregnancy surveillance – *n* (%)	12 (100.0)
Cesarean delivery – *n* (%)	5 (41.7)
Gestational age – median (IQR) (weeks)	40 (3)
Birth weight – mean ± SD (g)	3152 ± 930
Length at birth – mean ± SD (cm)	47.0 ± 2.8
HC at birth – mean ± SD (cm)	34.0 ± 1.7
Apgar score < 7 at 5 min	0 (0.0)
Endotracheal intubation – *n* (%)	0 (0.0)
Female sex – *n* (%)	11 (91.7)
Year of birth – *n* (%)	
[2005–2009]	4 (33.3)
[2010–2014]	2 (16.7)
[2015–2019]	3 (25.0)
[2020–2024]	3 (25.0)
Year of Diagnosis – *n* (%)	
[2010–2014]	3 (25.0)
[2015–2019]	4 (33.3)
[2020–2024]	5 (41.7)
Psychomotor development milestones	
Sit without support – median (IQR) (months)	13.5 (2.5)
Talk – median (IQR) (months)	15.0 (7.5)
Walk – median (IQR) (months)	20.0 (3.5)

*HC* head circumference, *IQR* interquartile range, *SD* standard deviation

In the cohort, sitting without support was achieved at a median age of 13.5 months (IQR 2.5), first words at 15 months (IQR 7.5), and walking without support at 20 months (IQR 3.5).

### Diagnosis

The median age at symptom onset was 15 months (IQR 15) and at first visit to our center was 28 months (IQR 18). The most common initial concern of caregivers was motor delay in five children (41.7%) and speech delay in four children (33.3%). Six patients (50.0%) had a first reported diagnosis of global developmental delay, four (33.3%) had a diagnosis of epilepsy, and three (25.0%) had a diagnosis of autism spectrum disorder.

The median age at suspected clinical diagnosis was 29 months (IQR 17) and at genetic confirmation was 35 months (IQR 20). The *MECP2* variants reported in the cohort can be seen in Table 3. Eight patients (66.7%) underwent whole genome sequencing, and four (33.3%) were diagnosed by single gene molecular testing.

**Table 3:** *MECP2* variants reported in the cohort

cDNA*	Protein	*n* (%)
c.634delG	p.Val212SerfsX36	2 (16.7)
c.1157_1197del41	p.Leu386HisfsX5	2 (16.7)
c.1051_1214del	p.Ser351fs	1 (8.3)
c.674 C > G	p.Pro225Arg	1 (8.3)
c.1138 G > A	p.Val380Met	1 (8.3)
c.916 C > T	p.Arg.306Cys	1 (8.3)
c.877dup	p.lle293AsnfsX38	1 (8.3)
c.808 C > T	p.Arg270X	1 (8.3)
c.433 C > T	p.Arg145Cys	1 (8.3)
c.842del	p.Gly281AlafsX20	1 (8.3)
c.695del	p.Gly232AlafsX16	1 (8.3)

^*^One female patient with classic RTT had two different variants of the *MECP2* gene (see page 1 of the Supplementary File for more details)

Based on the revised diagnostic criteria of the Rett Search Consortium [7], seven patients (58.3%) had been diagnosed with classical RTT. The main and supportive criteria reported in the cohort are shown in Table 4.

**Table 4:** Main and supportive criteria reported in the cohort

	*n* (%)
Main criteria	
Gait abnormalities	12 (100.0)
Stereotypic hand movements	11 (91.7)
Loss of acquired spoken language	10 (83.3)
Loss of acquired purposeful hand skills	6 (50.0)
Supportive criteria	
Abnormal muscle tone	12 (100.0)
Impaired sleep pattern	9 (75.0)
Scoliosis/kyphosis	7 (58.3)
Breathing disturbances when awake	6 (50.0)
Growth retardation	4 (33.3)
Bruxism when awake	4 (33.3)
Peripheral vasomotor disturbances	2 (16.7)
Small cold hands and feet	2 (16.7)
Inappropriate laughing/screaming spells	2 (16.7)
Diminished response to pain	1 (8.3)
Intense eye communication	1 (8.3)

**Fig. 1:**
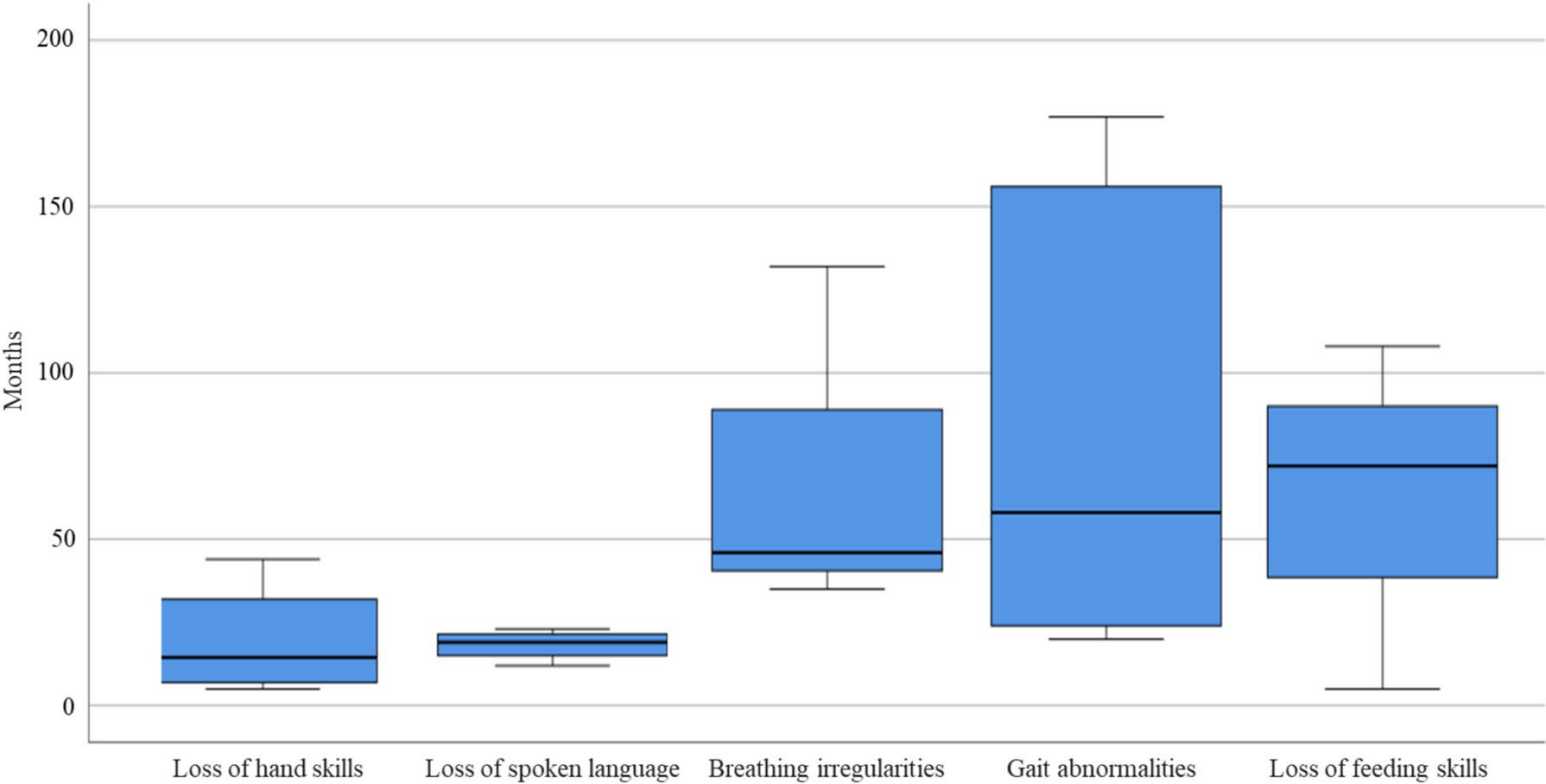
Time course of the onset of the symptoms

Hand skills were the first to be lost at a median age of 14.5 months (IQR 32), followed by loss of spoken language at a median age of 19 months (IQR 9). Figure 1 shows the time course of symptom onset.

Scoliosis and/or kyphosis were present in seven children (58.3%), with a median age at the diagnosis of 6 years (IQR 5). Growth retardation was present in four patients (33.3%), with a median age at the onset of 2 years (IQR 2).

The most common finding in neurological examination was gait abnormalities (present in all cases), including wide base gait (*n* = 6), apraxic gait (*n* = 4), walking on tiptoe (*n* = 1), and absence of gait (*n* = 1); stereotypic hand movements (*n* = 10); axial and appendicular ataxia (*n* = 5); spasticity of all four limbs (*n* = 4); hypotonia (*n* = 4); strabismus (*n* = 2); and flaccid tetraparesis (*n* = 1). No other cranial nerve involvement or pyramidal signs (hyperreflexia, extensor plantar responses, clonus) were noted.

### Medical comorbidities

The median number of medical comorbidities per child was three, with a maximum of five. One patient has no medical comorbidity: a 2-year-old girl diagnosed with classic RTT in December 2024. The majority of children were diagnosed with epilepsy (*n* = 9; 75.0%) and constipation (*n* = 8; 66.7%).

The median age at diagnosis of epilepsy was 2 years (IQR 2), and all patients with epilepsy underwent an electroencephalogram, which revealed developmental and epileptic encephalopathy and the following findings: slow basal rhythm with focal paroxysmal activity (*n* = 5), slow basal rhythm with generalized paroxysmal activity (*n* = 3), and Lennox-Gastaut syndrome (*n* = 1). Four of these patients (44.4%) had intractable epilepsy.

None of the patients had microcephaly at birth. However, during childhood, three patients (25.0%) developed microcephaly, with a median age at onset of 6 months (IQR 1).

Five children (41.7%) were wheelchair dependent, with a median age at onset of 6 years (IQR 8). Two children were also dependent on other medical devices or equipment. One child (the boy) started non-invasive respiratory support at 16 months of age, after a period of mechanical ventilation in the context of pneumonia, and had a gastrostomy placement at 26 months of age due to feeding difficulties after a long period of enteral tube feeding. The other child started on non-invasive respiratory support at the age of 17-year-old due to obstructive sleep apnea.

All patients were on at least three different therapies or support measures. The median number of therapies per child was six, with a maximum of eight. The most common therapies were physiotherapy (*n* = 12; 100.0%), followed by occupational and speech therapy (*n* = 11; 91.7%).

Eleven patients were on at least one chronic drug. The main class was anti-seizure medication (*n* = 9; 81.8%) followed by laxatives (*n* = 6; 54.5%). The median number of drugs per child was two, with a maximum of six. Among the nine patients diagnosed with epilepsy, the median number of antiepileptic drugs per child was one (minimum 1–maximum 3), and the most common drug was clobazam (*n* = 5; 55.6%), followed by valproic acid and levetiracetam (*n* = 3 each; 33.3%).

All comorbidities and therapies reported in the cohort are shown in Table 5.

**Table 5:** Comorbidities and therapies reported in the cohort

	*n* (%*)
Comorbidities	11 (91.7)
Epilepsy	9 (75.0)
Constipation	8 (66.7)
Microcephaly	3 (25.0)
Strabismus	2 (16.7)
Dependence on medical devices	5 (41.7)
Wheelchair	5 (41.7)
Non-invasive respiratory support	2 (16.7)
Gastrostomy placement	1 (8.3)
Therapies and support measures	12 (100.0)
Physiotherapy	12 (100.0)
Occupational therapy	11 (91.7)
Speech therapy	11 (91.7)
NEISC support	7 (58.3)
Hydrotherapy	6 (50.0)
Hippotherapy	6 (50.0)
Learning and inclusion measures	6 (50.0)
*Snoezelen* therapy	4 (33.3)
Alternative medicine	2 (16.7)
Canine-assisted therapy	1 (8.3)
Chronic drugs	11 (91.7)
Anti-seizure medication	9 (75.0)
Laxatives	6 (50.0)
Benzodiazepines	4 (33.3)
Antipsychotics	3 (25.0)
Muscle relaxant	1 (8.3)
Cannabinoid	1 (8.3)
Hormone	1 (8.3)
Vitamin	1 (8.3)

^*^All percentages refer to the total sample (*n* = 12)

*NEISC* National Early Intervention System in Childhood

### Clinical follow-up

The median follow-up of the cohort was six years (minimum 5 months–maximum 16 years), and the median current age of the children is 11 years (minimum 2 years–maximum 19 years). All patients had multidisciplinary follow-up in our center. The median number of medical specialties per child was two, with a maximum of five. The most common specialties were neuropediatrics and neurodevelopmental pediatrics (*n* = 9; 75.0%), followed by pediatric orthopedics (*n* = 5; 41.7%). One patient (8.3%) was referred to the palliative care team.

By the end of the study, only one child had died (8.3%): the 10-year-old boy with pneumonia complicated by sepsis and multiorgan failure.

## Discussion

The prevalence of RTT appears to have remained relatively stable over the 15 years of the study, with a slight rise of one additional case diagnosed in each successive 5-year period. The median age at diagnosis was 35 months (IQR 20) but appears to have decreased over the past 10 years, similar to trends observed in some other centers [8, 9, 19]. This may reflect increased awareness and knowledge of the disorder among health care system, society, educators, and other health care professionals, as well as increased access to genetic testing.

There was a clear predominance of females with RTT (91.7%), as it was expected. Nevertheless, and despite the small sample size of 12 patients, it was possible to identify one male child with atypical RTT. The genetic confirmation of his case was made after a neurodevelopment regression, and in the presence of the following clinical features: gait abnormalities, loss of acquired spoken language, abnormal muscle tone, scoliosis, breathing disturbances, growth retardation, peripheral vasomotor disturbances, and small cold hands and feet. Males with the condition often present with a more severe phenotype and poorer prognosis [1–3, 5, 7, 8, 10]. However, due to different MECP2 variants, males can experience varying degrees of clinical difficulty ranging from moderate intellectual disability to male RTT encephalopathy [12]. We present a case of RTT in a boy who experienced a worse outcome than the female cohort. He exhibited symptom onset at nine months (earlier than the median of 15 months for the cohort), required more medical devices (gastrostomy, non-invasive ventilation, and a wheelchair), and was the only reported death, occurring at age 10 due to complicated pneumonia. Indeed, respiratory illnesses and seizure-related conditions are the most common causes of death in RTT [20].

Molecular analysis in our study identified 11 different pathogenic variants in the *MECP2* gene, all of which were de novo spontaneous mutations. The most frequently identified were c.634delG and c.1157-1197del41, each found in two unrelated children. Several studies describe a correlation between mutation type and the disease phenotype [4, 5, 10, 21–25]; however, the presence of modifier mutations and random X-inactivation (also known as lyonization) in different cells of the body can influence the clinical expression of *MECP2* mutations [2–4].

It should be emphasized that, although we have included in our study only cases of RTT with mutations in the *MECP2* gene and despite the strong and proven association between both, the *MECP2* mutation is neither a necessary nor a sufficient criterion for the diagnosis of RTT. Its diagnosis is primarily clinical and, in all cases, relies on a regression of neurodevelopment [7]. According to the revised diagnostic criteria of the Rett Search Consortium from 2010 [7], we had seven cases of RTT with a classical phenotype and six cases with an atypical phenotype. Among the four major clinical criteria, all children presented with gait abnormalities, 91.7% with stereotypic hand movements (the first skill to be affected), 83.3% with language regression, and 50.0% with hand use regression. Among the minor supportive criteria, the most frequently observed in over half of the cases were abnormal muscle tone, impaired sleep pattern, and scoliosis/kyphosis.

Our study corroborates that females with RTT are generally born at term after an uncomplicated pregnancy and delivery. Grossly abnormal psychomotor development during the first 6 months of life is an exclusion criterion for classic RTT [7]. However, as reported by Einspieler et al., some nonspecific signs may be present during this period, including abnormal general movements, tongue protrusion, postural stiffness, asymmetric eye opening and closing, and abnormal finger movements [26, 27]. Although these signs are not unique to RTT, they may facilitate earlier clinical recognition and intervention [27]. Deceleration of head growth may also be present in the first 6 months of life [27, 28]. This clinical feature is no longer considered as a necessary criterion for diagnosis, as it is not found in all individuals with typical RTT. However, when present, it should alert clinicians to consider the possibility of this disease. Deceleration of head growth is distinct from microcephaly, but it can ultimately lead to its development. Microcephaly was present in only 25.0% of our cases, not a common comorbidity in this diagnosis [7, 29].

In accordance with the literature, the majority of children were also diagnosed with epilepsy (*n* = 9; 75.0%) and constipation (*n* = 8; 66.7%), which accounts for the high prescription rates of anti-seizure medication (*n* = 9; 75.0%) and laxatives (*n* = 6; 50.0%) [1, 6].

Our findings regarding epilepsy were consistent with those previously reported in the literature. Epilepsy has been reported in 60–80% of patients with RTT, with the highest frequency of seizures occurring between 2 and 10 years of age [30, 31]. The time of seizure onset has been associated with the prognosis of epilepsy, with early seizure onset being associated with more seizure types, intractable epilepsy, and status epilepticus [32]. Although all seizure types have been reported, complex partial and generalized tonic–clonic seizures are the most common seizure types in RTT [30, 33, 34]. In our cohort, 75.0% of patients were diagnosed with epilepsy and 44.4% of them had intractable epilepsy. The early onset of seizures, with a median age of 2 years, may be one of the possible explanations for the high percentage of intractable epilepsy in our cohort.

Given the significant prevalence of multisystemic manifestations and comorbidities in RTT, the crucial importance of a multidisciplinary approach and follow-up is emphasized. In our study, all patients were on at least three different therapies or support measures (with a median of six therapies per child) and received multidisciplinary follow-up. The most important therapies were physiotherapy, occupational, and speech therapies, in line with the literature, working together with the final goal of improving quality of life and providing essential support for both patients and their families [35].

Trofinetide (Dayblue®) is the first and currently the only approved treatment for RTT. However, it was only approved for use in the USA and Canada. In these countries, it can only be used in pediatric patients who are at least 2 years old and weigh at least 9 kg [36]. Gene therapy is undoubtedly a promising area of research. Several clinical trials are currently underway, although still in the early stages (phase 1 of the clinical trial), aiming to introduce a copy of the *MECP2* gene into neurons using a vector that is delivered into the body through intraventricular or intrathecal injection. The ultimate goal is to restore the normal function of the *MECP2* gene, thereby providing new hope for better disease control and possible significant changes in phenotype [4, 37].

The importance of our study is underscored by the fact that, in the past decade, no studies have been published on RTT in Portugal, and there is a lack of Portuguese research with such extensive long-term follow-up. The last Portuguese study on RTT was published in 2011 by Temudo et al. [22] and focused on the genotype–phenotype correlation, exploring RTT with and without detected *MECP2* mutations. Our study fills this gap, providing valuable data and insights that enhance the understanding of RTT within the Portuguese context and contributes to the global body of knowledge on this rare and very severe disease.

Our study has some limitations. On account of this study being restricted to a single center, these findings may be skewed and must be interpreted with caution. Due to its retrospective nature, diagnoses and comorbidities may have been underreported. However, we were able to obtain complete information in all patients.

In conclusion, despite the small number of patients, this study allows the characterization of a rare disease. Although core features are present, it demonstrates a significant expression of both classic and atypical RTT phenotypes associated with alterations in *MECP2* gene. This study also illustrates the complexity surrounding the multidisciplinary evaluation of these patients. The importance of maintaining structured clinical records and pursuing national and international collaborative projects in the field of this disease is emphasized in order to improve the understanding of disease and genotype-phenotype relationships.

## Supplementary Information

Below is the link to the electronic supplementary material.

Supplementary file 1 (PDF 243 KB)

## Data Availability

No datasets were generated or analysed during the current study.

## Abbreviations

MECP2: Methyl-CpG-binding protein 2
RTT: Rett syndrome
