# Supplementary material for: Clinical and functional outcomes in pediatric patients with Rett syndrome: a 15-year retrospective study
Source: Eur J Pediatr. 2025 Jul 3;184(7):465. doi: 10.1007/s00431-025-06291-6 (PMC12226632; doi:10.1007/s00431-025-06291-6)
Supplement: Supplementary file 1 — (PDF 243 KB) [file 431_2025_6291_MOESM1_ESM.pdf]

Appendices

Appendix I – Detailed patient description (Patient 1)

Year of birth / Age	Characteristics at birth	Diagnosis	Clinical characteristics		Follow-up
2006<br>/<br>19 years	**Gender:**<br>Female<br><br>**Gestational age:**<br>38 weeks<br><br>**Birth weight:**<br>3240 g<br><br>**Delivery:**<br>Cesarean	***MECP2* variant:**<br>c.1157-1197del41 (p.Leu386HisfsX5)<br>c.1138G>A (p.Val380Met)<br><br>**Age at symptom onset (1st symptom):**<br>18 months (loss of language)<br><br>**Age at genetic diagnosis:**<br>61 months<br><br>**RTT phenotype:**<br>Classic	**Main criteria:**<br>Gait abnormalities<br>Stereotypic hand movements<br>Loss of acquired spoken language<br>Loss of acquired purposeful hand skills<br><br>**Supportive criteria:**<br>Abnormal muscle tone<br>Impaired sleep pattern<br>Scoliosis/kyphosis<br>Breathing disturbances<br>Growth retardation<br>Peripheral vasomotor disturbances<br>Small cold hands and feet	**Comorbidities:**<br>Epilepsy<br>Constipation<br>Complete loss of gait<br>Microcephaly<br><br>**Medical equipment:**<br>Wheelchair (18 years old)<br>Non-invasive respiratory support (17 years old)	**Therapies and support measures:**<br>Physiotherapy<br>Occupational therapy<br>Speech therapy<br>Hippotherapy<br>NEISC support<br>Learning and inclusion measures<br><br>**Chronic drugs:**<br>Anti-seizure medication<br>Laxatives<br>Benzodiazepines

Appendix I (continuation) – Detailed patient description (Patient 2 and Patient 3)

Year of birth / Age	Characteristics at birth	Diagnosis	Clinical characteristics		Follow-up
2009 / 15 years	**Gender:**<br>Female<br><br>**Gestational age:**<br>41 weeks<br><br>**Birth weight:**<br>3065 g<br><br>**Delivery:**<br>Cesarean	***MECP2* variant:**<br>c.1157_1197del (p.Leu386HisfsX5)<br><br>**Age at symptom onset (1st symptom):**<br>36 months (loss of language)<br><br>**Age at genetic diagnosis:**<br>95 months<br><br>**RTT phenotype:**<br>Atypical	**Main criteria:**<br>Gait abnormalities<br>Stereotypic hand movements<br>Loss of acquired spoken language<br><br>**Supportive criteria:**<br>Abnormal muscle tone<br>Impaired sleep pattern<br>Scoliosis/kyphosis<br>Breathing disturbances<br>Growth retardation<br>Bruxism	**Comorbidities:**<br>Epilepsy<br>Complete loss of gait<br><br>**Medical equipment:**<br>Wheelchair (15 years old)	**Therapies and support measures:**<br>Physiotherapy<br>Occupational therapy<br>Speech therapy<br>Hippotherapy<br>NEISC support<br>Snoezelen therapy<br><br>**Chronic drugs:**<br>Anti-seizure medication<br>Antipsychotics
2009 / 15 years	**Gender:**<br>Female<br><br>**Gestational age:**<br>40 weeks<br><br>**Birth weight:**<br>3055 g<br><br>**Delivery:**<br>Vaginal	***MECP2* variant:**<br>c.634delG (p.Val212SerfsX36)<br><br>**Age at symptom onset (1st symptom):**<br>18 months (no sitting without support)<br><br>**Age at genetic diagnosis:**<br>26 months<br><br>**RTT phenotype:**<br>Classic	**Main criteria:**<br>Gait abnormalities<br>Stereotypic hand movements<br>Loss of acquired spoken language<br>Loss of acquired purposeful hand skills<br><br>**Supportive criteria:**<br>Abnormal muscle tone	**Comorbidities:**<br>Epilepsy<br><br>**Medical equipment:**<br>None	**Therapies and support measures:**<br>Physiotherapy<br>Occupational therapy<br>Speech therapy<br>Hydrotherapy<br>Hippotherapy<br>NEISC support<br><br>**Chronic drugs:**<br>Anti-seizure medication

NEISC, National Early Intervention System in Childhood

Appendix I (continuation) – Detailed patient description (Patient 4 and Patient 5)

Year of birth / Age	Characteristics at birth	Diagnosis	Clinical characteristics		Follow-up
2009 / 15 years	**Gender:**<br>Female<br><br>**Gestational age:**<br>40 weeks<br><br>**Birth weight:**<br>3660 g<br><br>**Delivery:**<br>Vaginal	***MECP2* variant:**<br>c.634delG (p.Val212SerfsX36)<br><br>**Age at symptom onset (1st symptom):**<br>18 months (lack of interest in objects)<br><br>**Age at genetic diagnosis:**<br>26 months<br><br>**RTT phenotype:**<br>Classic	**Main criteria:**<br>Gait abnormalities<br>Stereotypic hand movements<br>Loss of acquired spoken language<br>Loss of acquired purposeful hand skills<br><br>**Supportive criteria:**<br>Abnormal muscle tone<br>Impaired sleep pattern	**Comorbidities:**<br>Epilepsy<br>Constipation<br><br>**Medical equipment:**<br>None	**Therapies and support measures:**<br>Physiotherapy<br>Occupational therapy<br>Speech therapy<br>Hydrotherapy<br>Hippotherapy<br>NEISC support<br>Learning and inclusion measures<br><br>**Chronic drugs:**<br>Anti-seizure medication
2013 / 11 years	**Gender:**<br>Female<br><br>**Gestational age:**<br>30 weeks<br><br>**Birth weight:**<br>1235 g<br><br>**Delivery:**<br>Cesarean	***MECP2* variant:**<br>c.808C>T (p.Arg270X)<br><br>**Age at symptom onset (1st symptom):**<br>18 months (hypotonia)<br><br>**Age at genetic diagnosis:**<br>39 months<br><br>**RTT phenotype:**<br>Atyical	**Main criteria:**<br>Gait abnormalities<br>Stereotypic hand movements<br><br>**Supportive criteria:**<br>Abnormal muscle tone<br>Impaired sleep pattern<br>Scoliosis/kyphosis<br>Breathing disturbances<br>Bruxism	**Comorbidities:**<br>Epilepsy<br>Constipation<br><br>**Medical equipment:**<br>None	**Therapies and support measures:**<br>Physiotherapy<br>Occupational therapy<br>Speech therapy<br>Hydrotherapy<br><br>**Chronic drugs:**<br>Anti-seizure medication<br>Laxatives

NEISC, National Early Intervention System in Childhood

Appendix I (continuation) – Detailed patient description (Patient 6)

Year of birth / Age	Characteristics at birth	Diagnosis	Clinical characteristics		Follow-up
2013 / Not applicable	**Gender:**<br>Male<br><br>**Gestational age:**<br>40 weeks<br><br>**Birth weight:**<br>3500 g<br><br>**Delivery:**<br>Vaginal	***MECP2* variant:**<br>c.842del (p.Gly281AlafsX20)<br><br>**Age at symptom onset (1st symptom):**<br>9 months (loss of language)<br><br>**Age at genetic diagnosis:**<br>26 months<br><br>**RTT phenotype:**<br>Atypical	**Main criteria:**<br>Gait abnormalities<br>Loss of acquired spoken language<br><br>**Supportive criteria:**<br>Abnormal muscle tone<br>Scoliosis/kyphosis<br>Breathing disturbances<br>Growth retardation<br>Peripheral vasomotor disturbances<br>Small cold hands and feet	**Comorbidities:**<br>Epilepsy<br>Constipation<br>Complete loss of gait<br>Microcephaly<br><br>**Medical equipment:**<br>Wheelchair (since birth)<br>Non-invasive respiratory support (16 months old)<br>Gastrostomy placement (26 months old)	**Therapies and support measures:**<br>Physiotherapy<br>Speech therapy<br>Alternative medicine<br><br>**Chronic drugs:**<br>Anti-seizure medication<br>Laxatives<br>Benzodiazepines<br>Muscle relaxant<br>Cannabinoid<br>Vitamin<br><br>**Age and cause of death:**<br>10 years old<br>Pneumonia complicated by sepsis and multiorgan failure

7

Appendix I (continuation) – Detailed patient description (Patient 7 and Patient 8)

Year of birth / Age	Characteristics at birth	Diagnosis	Clinical characteristics		Follow-up
2015<br>/<br>9 years	**Gender:**<br>Female<br><br>**Gestational age:**<br>41 weeks<br><br>**Birth weight:**<br>3555 g<br><br>**Delivery:**<br>Vaginal	***MECP2* variant:**<br>c.1051_1214del (p.Ser351fs)<br><br>**Age at symptom onset (1st symptom):**<br>12 months (loss of language)<br><br>**Age at genetic diagnosis:**<br>30 months<br><br>**RTT phenotype:**<br>Classic	**Main criteria:**<br>Gait abnormalities<br>Stereotypic hand movements<br>Loss of acquired spoken language<br>Loss of acquired purposeful hand skills<br><br>**Supportive criteria:**<br>Abnormal muscle tone<br>Impaired sleep pattern<br>Scoliosis/kyphosis	**Comorbidities:**<br>Epilepsy<br>Constipation<br>Complete loss of gait<br>Strabismus<br><br>**Medical equipment:**<br>Wheelchair (7 years old)	**Therapies and support measures:**<br>Physiotherapy<br>Occupational therapy<br>Speech therapy<br>Hydrotherapy<br>Hippotherapy<br>Learning and inclusion measures<br>*Snoezelen* therapy<br>Canine-assisted therapy<br><br>**Chronic drugs:**<br>Anti-seizure medication<br>Laxatives
2017<br>/<br>7 years	**Gender:**<br>Female<br><br>**Gestational age:**<br>41 weeks<br><br>**Birth weight:**<br>3040 g<br><br>**Delivery:**<br>Cesarean	***MECP2* variant:**<br>c.433C>T (p.Arg145Cys)<br><br>**Age at symptom onset (1st symptom):**<br>21 months (no gait)<br><br>**Age at genetic diagnosis:**<br>41 months<br><br>**RTT phenotype:**<br>Classic	**Main criteria:**<br>Gait abnormalities<br>Stereotypic hand movements<br>Loss of acquired spoken language<br>Loss of acquired purposeful hand skills<br><br>**Supportive criteria:**<br>Abnormal muscle tone<br>Impaired sleep pattern<br>Scoliosis/kyphosis	**Comorbidities:**<br>Epilepsy<br>Constipation<br>Complete loss of gait<br>Microcephaly<br>Strabismus<br><br>**Medical equipment:**<br>Wheelchair (6 years old)	**Therapies and support measures:**<br>Physiotherapy<br>Occupational therapy<br>Speech therapy<br>Hydrotherapy<br>Hippotherapy<br>Learning and inclusion measures<br>Alternative medicine<br><br>**Chronic drugs:**<br>Anti-seizure medication<br>Benzodiazepines

Appendix I (continuation) – Detailed patient description (Patient 9 and Patient 10)

Year of birth / Age	Characteristics at birth	Diagnosis	Clinical characteristics	Follow-up
2019 / 5 years	**Gender:**<br>Female<br><br>**Gestational age:**<br>40 weeks<br><br>**Birth weight:**<br>3430 g<br><br>**Delivery:**<br>Cesarean	***MECP2* variant:**<br>c.877dup (p.lle293AsnfsX38)<br><br>**Age at symptom onset (1st symptom):**<br>18 months (headbanging)<br><br>**Age at genetic diagnosis:**<br>46 months<br><br>**RTT phenotype:**<br>Classic	**Main criteria:**<br>Gait abnormalities<br>Stereotypic hand movements<br>Loss of acquired spoken language<br>Loss of acquired purposeful hand skills<br><br>**Comorbidities:**<br>Constipation<br><br>**Supportive criteria:**<br>Abnormal muscle tone<br>Breathing disturbances<br>Inappropriate laughing/ screaming spells<br><br>**Medical equipment:**<br>None	**Therapies and support measures:**<br>Physiotherapy<br>Occupational therapy<br>Speech therapy<br>Learning and inclusion measures<br>*Snoezelen* therapy<br><br>**Chronic drugs:**<br>Laxatives<br>Antipsychotics
2020 / 4 years	**Gender:**<br>Female<br><br>**Gestational age:**<br>38 weeks<br><br>**Birth weight:**<br>3770 g<br><br>**Delivery:**<br>Cesarean	***MECP2* variant:**<br>c.674C>G (p.Pro225Arg)<br><br>**Age at symptom onset (1st symptom):**<br>24 months (muscle weakness)<br><br>**Age at genetic diagnosis:**<br>36 months<br><br>**RTT phenotype:**<br>Atypical	**Main criteria:**<br>Gait abnormalities<br>Stereotypic hand movements<br>Loss of acquired spoken language<br><br>**Comorbidities:**<br>Epilepsy<br><br>**Supportive criteria:**<br>Abnormal muscle tone<br>Impaired sleep pattern<br>Scoliosis/kyphosis<br>Growth retardation<br>Bruxism<br><br>**Medical equipment:**<br>None	**Therapies and support measures:**<br>Physiotherapy<br>Occupational therapy<br>Speech therapy<br>NEISC support<br>Learning and inclusion measures<br>*Snoezelen* therapy<br><br>**Chronic drugs:**<br>Anti-seizure medication

NEISC, National Early Intervention System in Childhood

Appendix I (continuation) – Detailed patient description (Patient 11 and Patient 12)

Year of birth / Age	Characteristics at birth	Diagnosis	Clinical characteristics	Follow-up
2020<br>/<br>4 years	**Gender:**<br>Female<br><br>**Gestational age:**<br>38 weeks<br><br>**Birth weight:**<br>3085 g<br><br>**Delivery:**<br>Vaginal	***MECP2* variant:**<br>c.916C>T (p.Arg306Cys)<br><br>**Age at symptom onset (1st symptom):**<br>5 months (hand tremor)<br><br>**Age at genetic diagnosis:**<br>17 months<br><br>**RTT phenotype:**<br>Atypical	**Main criteria:**<br>Gait abnormalities<br>Stereotypic hand movements<br>Loss of acquired spoken language<br><br>**Comorbidities:**<br>Constipation<br><br>**Supportive criteria:**<br>Abnormal muscle tone<br>Impaired sleep pattern<br>Breathing disturbances<br>Bruxism<br>Inappropriate laughing/ screaming spells<br>Intense eye communication<br><br>**Medical equipment:**<br>None	**Therapies and support measures:**<br>Physiotherapy<br>Occupational therapy<br>Hydrotherapy<br>NEISC support<br><br>**Chronic drugs:**<br>Laxatives<br>Benzodiazepines<br>Antipsychotics<br>Hormone

Year of birth / Age	Characteristics at birth	Diagnosis	Clinical characteristics	Follow-up
2023<br>/<br>2 years	**Gender:**<br>Female<br><br>**Gestational age:**<br>40 weeks<br><br>**Birth weight:**<br>3190 g<br><br>**Delivery:**<br>Vaginal	***MECP2* variant:**<br>c.695del (p.Gly232AlafsX16)<br><br>**Age at symptom onset (1st symptom):**<br>9 months (regression)<br><br>**Age at genetic diagnosis:**<br>23 months<br><br>**RTT phenotype:**<br>Classic	**Main criteria:**<br>Gait abnormalities<br>Stereotypic hand movements<br>Loss of acquired spoken language<br>Loss of acquired purposeful hand skills<br><br>**Comorbidities:**<br>None<br><br>**Supportive criteria:**<br>Abnormal muscle tone<br>Impaired sleep pattern<br><br>**Medical equipment:**<br>None	**Therapies and support measures:**<br>Physiotherapy<br>Occupational therapy<br>Speech therapy<br>NEISC support<br><br>**Chronic drugs:**<br>None

NEISC, National Early Intervention System in Childhood
